# Multicomponent Dietary Supplementation: Impact on Tear Secretion and Ocular Surface Inflammation in Dry Eye Syndrome Patients

**DOI:** 10.3390/antiox14010103

**Published:** 2025-01-16

**Authors:** Shih-Chien Huang, Yen-Ping Lei, Min-Chien Hsiao, Yu-Kai Hsieh, Quei-Ping Tang, Connie Chen, Min-Yen Hsu

**Affiliations:** 1 Department of Nutrition, Chung Shan Medical University, Taichung City 402, Taiwan; schuang@csmu.edu.tw; 2 Department of Nutrition, Chung Shan Medical University Hospital, Taichung City 402, Taiwan; 3 Department of Nursing, National Yang Ming Chiao Tung University, Hsinchu City 300, Taiwan; yplei@nycu.edu.tw; 4 Department of Medical Education, Changhua Christian Hospital, Changhua City 500, Taiwan; 184984@cch.org.tw; 5 School of Medicine, Taipei Medical University, Taipei City 110, Taiwan; b101111038@tmu.edu.tw; 6 Department of Nutrition, Wei Gong Memorial Hospital, Toufen City 351, Taiwan; 046346@tool.caaumed.org.tw; 7 Department of Ophthalmology, Chung Shan Medical University Hospital, Taichung City 402, Taiwan; cconnie7@csmu.edu.tw; 8 Department of Optometry, Chung Shan Medical University, Taichung City 402, Taiwan; 9 Institute of Optometry, Chung Shan Medical University, Taichung City 402, Taiwan; 10 School of Medicine, Chung Shan Medical University, Taichung City 402, Taiwan

**Keywords:** dry eye syndrome, inflammation, oxidative stress, fish oil, lutein, zeaxanthin

## Abstract

Dry eye syndrome (DES) is a prevalent ocular condition characterized by tear film instability, inflammation, and discomfort, affecting millions worldwide. DES is related to oxidative stress imbalance and ocular surface inflammation, which are important factors in the development of the condition. Recent studies have demonstrated that fish oil, lutein, and zeaxanthin possess anti-inflammatory and antioxidant properties. This study investigated the efficacy of a multicomponent dietary supplement in improving tear secretion and mitigating ocular surface inflammation in patients with DES. It was an open-label intervention trial. In total, 52 participants were randomly assigned to control (*n* = 23) and supplement (45 mg/day eicosapentaenoic acid, 30 mg/day docosahexaenoic acid, 30 mg/day lutein, and 1.8 mg/day zeaxanthin; *n* = 29) groups for 12 weeks. The participants were evaluated using Schirmer’s test and the ocular surface disease index (OSDI) as ocular surface parameters. Moreover, blood or tear oxidative stress, antioxidant capacities, and tear inflammatory indicators were measured at weeks 0 and 12. The results indicated a significant increase in tear secretion and a significant reduction in OSDI scores in the supplement group. Additionally, inflammatory markers, such as interleukin (IL)-6 and IL-8, significantly decreased after the intervention. However, the OSDI of the supplement group significantly improved by 6.60 points (β = −6.60, *p* = 0.01). These findings support the potential of targeted nutritional supplementation as a safe and effective strategy for alleviating DES symptoms, offering an alternative to conventional treatments that exclusively focus on symptom management. This study highlights the role of specific nutrients in modulating tear production and inflammation, thereby providing a foundation for dietary approaches to DES treatment. Future research should explore the long-term benefits of such interventions and their impact on overall ocular health.

## 1. Introduction

Dry eye syndrome (DES), a multifactorial disease of the ocular surface, is primarily characterized by tear film instability. This instability leads to increased tear film osmolarity and ocular surface inflammation or damage, resulting in discomfort [1]. DES significantly impacts patients’ quality of life [2]. As of 2021, the global prevalence of DES was estimated at 29.5%. Notably, Africa exhibited the highest regional prevalence rate (47.9%), followed by West Asia (29.0%); East Asia (19.4%); and Europe, South America, and Oceania (13.7–14.9%). Taiwan, Japan, and South Korea have reported rates of approximately 30.0% [3]. In Taiwan, approximately 25% of the population suffers from DES, and the incidence is increasing annually [4].

DES potentially arises from a multitude of single or multiple risk factors, encompassing personal attributes (e.g., age, post-menopausal status, and contact lens use), environmental conditions (e.g., prolonged eye fixation without adequate blinking), clinical diseases (e.g., autoimmune and chronic diseases), medications (e.g., psychiatric drugs, antiviral medications, beta blockers, and diuretics), and ocular surgeries (e.g., laser and cataract operations). These factors have been identified and discussed in various studies [5,6,7].

Inflammation is a key mechanism underlying the pathogenesis of DES. Research has demonstrated that in DES, epithelial cells release relatively high concentrations of chemokines and cytokines, notably tumor necrosis factor-alpha (TNF-α), interleukin (IL)-1β, IL-6, and IL-8 [8,9]. Among these, IL-6 and TNF-α have been identified as critical inflammatory markers in DES, contributing to lacrimal gland cell apoptosis and reduced tear production [10]. Patients with DES may develop an adaptive immune response leading to chronic inflammation [11]. This cytokine-mediated process affects sensory neurons and cytokine alterations, resulting in decreased lacrimal secretion and associated ocular surface discomfort [12,13].

Oxidative stress is another crucial mechanism contributing to the exacerbation of DES. Prolonged exposure of the ocular surface to factors such as ultraviolet radiation, air pollution, hormonal changes, and bacterial infections potentially induces the overproduction of reactive oxygen species (ROS). When antioxidant capabilities are insufficient or imbalanced, ROS can directly damage the ocular glands, leading to epithelial and goblet cell apoptosis or harm to corneal nerves, thereby diminishing tear secretion quality and tear film stability [14]. ROS also trigger inflammatory responses, rendering their overproduction a key factor in the DES cycle [15].

Clinically, artificial tears are commonly used to manage DES, aiming to increase moisture and prevent tear evaporation; however, they merely alleviate symptoms temporarily [16]. Researchers suggest that intense pulsed light therapy significantly improves DES symptoms; nevertheless, it is considered unsafe for upper-eyelid treatment, requires annual or biennial sessions, and is costly [17]. Autologous serum eye drops have recently emerged, and they are made from the patient’s own blood and cellular components to mimic the biologically active nutrients in natural tears, such as vitamins A and C, lysozyme, and immunoglobulins [18,19]. However, they are not suitable for patients with autoimmune disease, are prone to microbial contamination, and require refrigeration [20]. Additionally, the Food and Drug Administration has approved anti-inflammatory drugs such as cyclosporine A and lifitegrast 5% for DES; while they can improve symptoms, their long-term use may cause side effects such as conjunctival hyperemia, secretions, foreign body sensation, and decreased vision [16,21]. Consequently, dietary supplements for eye health have actively been explored and are considered safer for long-term use [22].

An in vitro study found omega-3 fatty acids, particularly eicosapentaenoic acid (EPA) and docosahexaenoic acid (DHA), to significantly enhance cell viability and reduce inflammation in corneal epithelial cells [23]. Numerous studies have indicated that high doses of fish oil, specifically EPA (1500–2000 mg) plus DHA (1000–1050 mg) daily for 3–6 months, potentially improve tear film break-up time, tear secretion, and the ocular surface disease index (OSDI) [24,25]. In contrast, low-dose fish oil supplementation often uses a compound formula to enhance efficacy. A study administered a high-dose fish oil compound (EPA 1050 mg + DHA 127.5 mg/day), which also included vitamins (A, C, and E), zinc, magnesium, copper, selenium, tyrosine, and cysteine, to patients with DES for 3 months. This regimen significantly increased tear secretion and tear film break-up time compared with the control [24]. Another study administered a fish oil compound (EPA 45 mg + DHA 700 mg/day), which also contained vitamins (A, C, and E), zinc, copper, and selenium, to patients with DES for 3 months, resulting in a significant reduction in inflammatory factors IL-1β, IL-6, and IL-8 [26].

Lutein and zeaxanthin, widely recognized as beneficial for eye health, can absorb 40–90% of incident blue light, thus protecting the retina from photodamage and reducing light scattering [27]. Their antioxidant properties help scavenge free radicals and enhance overall antioxidative capacity, preventing oxidative damage [28,29]. Furthermore, their anti-inflammatory properties are significant, as inflammation is a key pathogenic mechanism underlying several ocular diseases. Lutein and zeaxanthin can prevent oxidative stress-induced cytokine increase and regulate the expression of inflammation-related genes [30]. Research has demonstrated that administering lutein (20 mg/day) and zeaxanthin (2 mg/day) to patients with DES for 3 months significantly improves tear film break-up time and tear meniscus height [22]. Another study providing lutein (20 mg/day) and zeaxanthin (4 mg/day) for 8 weeks resulted in significant improvements in tear film break-up time, tear secretion, the OSDI, tear osmolarity, and matrix metallopeptidase-9 (MMP-9) levels [31]. These benefits may be attributed to lutein’s ability to inhibit IL-6 secretion by epithelial cells through the nuclear factor kappa B signaling pathway, highlighting its anti-inflammatory potential [32].

The incidence of DES has been increasing annually and is affecting younger populations [4]. However, the long-term health effects of high-dose fish oil supplementation are concerning. Moreover, the efficacy of dietary supplements containing a combination of fish oil, lutein, and zeaxanthin in ameliorating inflammation and oxidative stress and enhancing antioxidant capacity is yet to be elucidated. Therefore, this study aimed to investigate the effects of 12-week supplementation with a compound containing fish oil, lutein, and zeaxanthin on symptoms, oxidative stress, antioxidant capacity, and inflammation in patients with DES.

## 2. Methods

### 2.1. Study Design and Sample Size Determination

This study was an open-label intervention trial. The participants were randomly assigned to control and supplement (45 mg/day EPA, 30 mg/day DHA, 30 mg/day lutein, and 1.8 mg/day zeaxanthin) (Far East Bio-Tec Co., Ltd., Taipei City, Taiwan) groups. Supplementation was administered for 12 weeks. Each participant had two visits (weeks 0 and 12) throughout the study period. To assure compliance with the intervention, the participants were asked to return their bottles for capsule counts. In addition, the study co-executor made phone calls to remind each participant to take the capsules every week during the intervention period. A participant would be excluded if their compliance was <80%. In a previous study, the supplement group exhibited a significant increase in tear break-up time (TBUT) of 4.6 s (*p* < 0.05) compared with the control group [33]. We subsequently calculated the sample size based on the detection of a significant 4.6 s increase between two groups with a power of 80% and a two-sided test with an α value of 0.05. A total of 42 patients were required to match the calculation criteria. The final recruitment number was 52 patients, exceeding our original calculation.

### 2.2. Participants

**Figure 1:**
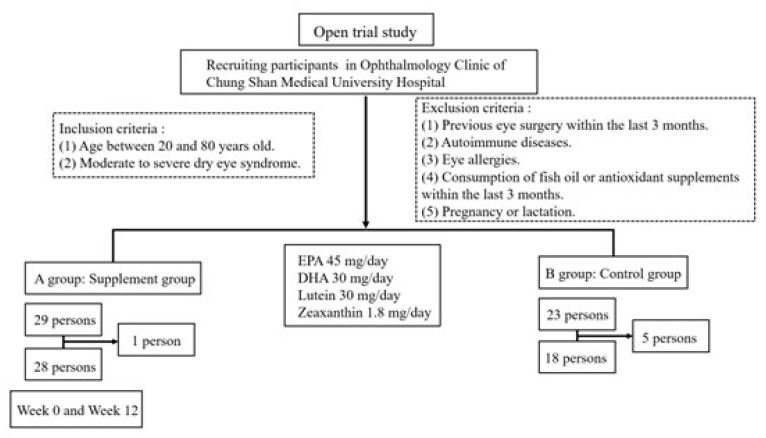
Clinical trial flowchart.

In total, 60 participants were initially enrolled at baseline from the Ophthalmology Outpatient Clinic of Chung Shan Medical University Hospital, Taiwan. The inclusion criteria were as follows: (1) age between 20 and 80 years and (2) the presence of moderate or severe DES. The exclusion criteria were as follows: (1) ophthalmic surgery within the preceding 3 months, (2) autoimmune disease, (3) ocular allergy, (4) the consumption of fish oil or antioxidant supplements within the preceding 3 months, and (5) pregnancy or lactation. The participant enrollment process is illustrated in Figure 1. A total of 8 participants withdrew owing to an inability to attend follow-up appointments and personal reasons, resulting in a final sample of 52 participants. The total dropout rate was 13.3%. This study was approved by the Institutional Review Board (IRB) of Chung Shan Medical University Hospital (IRB CSMUH No. CSI-20199). Each patient signed an informed consent form prior to participating, and the informed consent process was consistent with the principles of the Declaration of Helsinki.

### 2.3. Data Collection and Measurements

Data regarding each participant’s age, gender, self-reported DES symptoms, ophthalmic condition inquiries (high myopia: −5.00 D or more; high astigmatism: −1.50 D or more; past eye surgeries), medication usage, and lifestyle habits were collected. Additionally, the OSDI questionnaire was administered.

### 2.4. Schirmer Test

**Figure 2:**
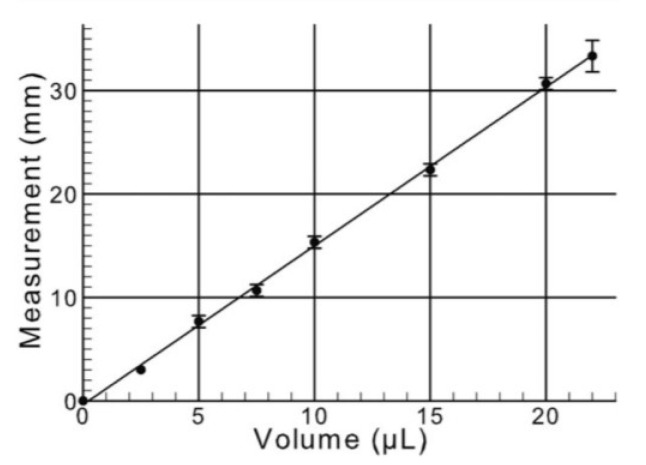
Graphical representation of tear volume measurements using Schirmer strips.

The Schirmer test is a common clinical method used to assess tear secretion. The procedure was repeated twice using filter paper strips placed inside the lower eyelid. After 5 min, each strip was removed, and the length of the moist section was measured to ascertain the amount of moisture absorbed. The conversion volume has been described in a previous study [34], as shown in Figure 2.

### 2.5. Ocular Surface Analyzer (OSA)

Dry eye analysis was performed using a non-invasive SBM Sistemi OSA to examine the tear film and meibomian glands. This non-contact method avoids errors resulting from contact irritation or fluorescein use. The analyzer automatically focuses and records data for quantitative analysis. Researchers have demonstrated that OSAs accurately assess tear film function, providing valuable insights into the clinical diagnosis of DES [34]. The parameters measured included non-invasive TBUT (NIBUT), lipid layer thickness (LLT), tear meniscus height (TMH), and meibomian gland loss (MG loss %).

### 2.6. Dietary Assessment

To ensure that the patients were maintaining their usual dietary intake, they had to complete 24 h dietary recalls at weeks 0 and 12. Nutritional composition was calculated using Nutritional composition was calculated using Nutritionist Pro™ software v7.9 (Nutrition Butler Enhanced Edition, E-Kitchen Business Corporation, Taichung City, Taiwan, 2002), and the nutrient database was based on the Taiwan food composition table (Department of Health, 2021).

### 2.7. Blood and Tear Collection

Fasting blood samples were drawn on the designated day. Blood specimens were collected in vacutainer tubes (Becton Dickinson, Rutherford, NJ, USA). Tear samples were collected using filter paper strips and diluted in 330 μL of phosphate-buffered saline. The original concentration of the tears was calculated based on the dilution [35,36].

### 2.8. Oxidative Stress and Total Antioxidant Capacity Evaluation Using Blood and Tear Specimens

To evaluate oxidative stress and antioxidant capacity, plasma and tear malondialdehyde (MDA) levels were assessed as indicators of oxidative stress. The plasma MDA concentration was measured along with thiobarbituric acid reactive substances at excitation and emission wavelengths of 515 and 555 nm, respectively, using a fluorescence spectrophotometer [37]. Tear MDA levels were measured using a commercial competitive enzyme-linked immunosorbent assay kit (Arigo, Hsinchu, Taiwan) [38]. Plasma and tear Trolox equivalent antioxidant capacity (TEAC) was analyzed using a previously described method [39].

### 2.9. Multiplex Cytokine Analysis

Tear samples were tested using the Bio-Rad Bio-Plex^®^ Pro Human Cytokine 27-Plex Assay on the Bio-Rad MAGPIX™ Multiplex Reader for the following inflammatory cytokines: IL-1β, IL-2, IL-6, IL-8, IL-17A, and TNF-α. Data were acquired using the Bio-Plex Array Reader System 200 (Bio-Rad, Hercules, CA, USA). The lower limits of quantitation (LOQs) for each cytokine are as follows: IL-1β: 0.00029 pg/μL; IL-2: 0.00129 pg/μL; IL-6: 0.00038 pg/μL; IL-8: 0.00085 pg/μL; IL-17α: 0.00244 pg/μL; and TNF-α: 0.00333 pg/μL.

### 2.10. Statistical Analysis

All data were analyzed using SigmaPlot software (version 12.5; Systat Software, San Jose, CA, USA) and presented on an intention-to-treat basis. A Shapiro–Wilk test was performed to determine normality. Between-group differences in demographic characteristics and experimental data were evaluated for significance using Student’s *t*-test or the Mann–Whitney rank-sum test. The χ^2^ or Fisher’s exact test was used to analyze categorical variables. Paired comparisons were performed using the paired t-test or Wilcoxon signed-rank test to examine changes in outcomes before and after the intervention. Partial Spearman’s correlation analysis was used to assess the association of changes in blood and tear oxidative stress, antioxidant capacity, and inflammatory factors with DES, after adjusting for potential confounders. Multiple linear regression analyses, with or without dietary supplementation as the dependent variable, were used to examine the association of changes in blood and tear oxidative stress, antioxidant capacity, and cytokines with DES, after adjusting for potential confounders. Statistical significance was set at *p* < 0.05.

## 3. Results

### Participant Demographics and Self-Assessment of Dry Eye Symptoms

This study enrolled 52 participants (29 participants in the supplement group and 23 in the control group). Their mean age was 58.0 years, with a range of 39–80 years. The compliance of the supplement group was 96.6 ± 5.4%, and no participants reflected any tolerance after taking the supplement. No significant differences in age, sex, or the proportion of participants with severe myopia or severe astigmatism were noted between the groups. However, the supplement group had a significantly greater proportion of participants who had undergone previous eye-related surgery than the control group. The self-assessment of dry eye symptoms revealed that dry eyes (80.8%) constituted the greatest proportion of symptoms, followed by eye fatigue (78.8%) and blurred or worsened vision (75.0%). No significant differences in various self-reported dry eye symptoms occurred between the two groups (Table 1).

The use of medication and lifestyle habits of participants before and after the intervention are shown in Table 2. Most participants did not use anti-inflammatory drugs. Regarding lifestyle habits, a significant difference in the average daily sleep duration was observed between the two groups, both before and after the intervention. However, no significant differences in other lifestyle habits were found between the groups.

Table 3 shows the results of DES-related indicators before and after the intervention. After the intervention, the tear secretion volume significantly increased by 30% in the left eye of the supplement group. Furthermore, the OSDI not only improved compared with that before the intervention but was also significantly lower than that of the control group. Post-intervention NIBUT significantly decreased in both the control and supplement groups (right eye) compared with that before the intervention. The degree of MG loss in the right eye of the supplement group significantly increased compared with that before the intervention.

No significant change in calorie intake occurred in either group before and after the intervention. Regarding macronutrients, protein intake significantly increased in the control group after the intervention, while a significant increase in fat intake occurred in the supplement group. Additionally, the control group exhibited a significantly higher lipid intake and lower carbohydrate intake than the supplement group, and fat intake in both groups ranged from 31% to 39% (Table 4).

No significant differences in tear TEAC levels were noted between the two groups or from week 0 to week 12. Regarding oxidative stress, plasma MDA levels significantly decreased, whereas tear fluid MDA levels significantly increased in the control group at week 12. In contrast, in the supplement group, no significant changes in oxidative stress manifested in either blood or tears from week 0 to week 12. After 12 weeks, the levels of tear cytokines, namely, IL-6 and IL-8, significantly decreased compared with those at week 0, and IL-6 also significantly decreased compared with that in the control group at week 12. Additionally, the post-intervention IL-17A concentration significantly increased in the control group at week 12 (Table 5).

We subsequently performed partial Spearman’s correlation coefficient analyses to assess the association of changes in oxidative stress indicators, antioxidant activity, and inflammatory responses with changes in DES indicators, after adjusting for age, gender, and menopausal status (Table 6). Tear secretion changes negatively correlated with changes in tear TEAC and MDA levels (right eye). Plasma TEAC changes negatively correlated with changes in the meniscus height (left eye) and OSDI of tears. In addition, the results indicated that alterations in tear secretion were inversely related to IL-1β (right eye) and IL-2, IL-8, and TNF-α (both eyes) levels. IL-17A changes were negatively associated with NIBUT (right eye) and TMH (left eye). Furthermore, the degree of MG loss exhibited a positive relationship with IL-6 (left eye) and IL-8 (left eye).

To ascertain whether supplement intake influenced dry eye-related indicators, oxidative stress indicators, antioxidant activity, and inflammatory responses, we conducted multiple linear regression analyses. Supplement intake was the dependent variable, and the models were adjusted for age, gender, and menopausal status. The results are presented in Table 7.

The findings revealed a significant improvement in the OSDI and an elevation in plasma TEAC levels following supplementation. After adjusting for potential confounders, an association was established between supplementation and OSDI improvement; however, the effect on plasma antioxidant capacity was not evident.

## 4. Discussion

After 12-week supplementation, the supplement group exhibited a significant increase in tear secretion and a significant reduction in tear inflammatory markers. This observation is consistent with the crucial role of inflammation in the pathogenesis of DES. Ocular surface epithelial cells are exposed to oxidative stress [40,41], and the innate immune response is triggered, leading to the release of TNF-α, IL-1β, and IL-6, which induce inflammation [42]. IL-6 stimulates the synthesis and release of acute-phase proteins, leading to the secretion of the pro-inflammatory cytokine IL-17, which induces apoptosis in lacrimal gland cells, thereby reducing tear production. Numerous clinical studies have confirmed a negative correlation between tear secretion and IL-6 and TNF-α [43,44]. Furthermore, inflammation or prolonged dryness of the ocular surface triggers a significant release of IL-8, which attracts neutrophil migration [10,45]. A previous study confirmed that increased tear film inflammation elevates ROS, leading to reduced tear secretion [46]. This study observed significantly negative correlations among changes in tear secretion, changes in tear MDA and TEAC levels, and inflammatory markers.

EPA and DHA have been shown to inhibit oxidative stress and mitigate the production of inflammatory mediators [47]. Lutein and zeaxanthin exhibit ROS scavenging properties [48]. Studies indicate that lutein can decrease the sensitivity of cell membranes to oxidative damage [49] and improve inflammation [50]. Clinical research has demonstrated that a 3-month supplementation regimen can significantly reduce tear IL-6 levels compared with the control [26] or increase tear secretion [24]. A 12-week supplementation regimen involving a combination of lutein, zeaxanthin, and curcumin significantly increased tear secretion in patients with DES [22]. Our findings are consistent with those obtained in the above studies, indicating that compound supplements significantly ameliorate inflammation. Notably, IL-17A significantly increased in the control group.

Additionally, we observed a significant increase in oxidative stress in the tears of the control group after 12 weeks. This finding is consistent with previous research that highlights how the disruption of redox homeostasis in DES contributes to the creation of a localized oxidative environment within the tear film [14]. Furthermore, both plasma antioxidant capacity and oxidative stress were significantly reduced in the control group. This paradoxical observation may reflect the complex role oxidative stress plays in the clinical progression of DES. The increase in oxidative stress could lead to the depletion of antioxidant defenses, as the body attempts to counteract the damaging effects of oxidative stress. Such depletion may represent an adaptive response to chronic oxidative stress, where cellular protective mechanisms engage antioxidants to neutralize harmful free radicals. As Liguori et al. [51] suggest, oxidative metabolism produces ROS, which further exacerbate oxidative stress. This feedback mechanism, although initially protective, may ultimately result in a reduction in both antioxidant capacity and oxidative stress markers in plasma. Moreover, additional factors such as lifestyle changes, environmental exposures, and dietary influences may further modulate oxidative and antioxidant systems, contributing to the complexity of the response. Future research should explore these variables and their intricate interactions to provide a more comprehensive understanding of the mechanisms driving DES progression and its associated oxidative stress. However, no changes in oxidative stress and antioxidant capacity indicators were observed in the supplement group, possibly because of the compound supplement providing sufficient antioxidant capacity to balance oxidants and antioxidants, thereby attenuating the potential for further deterioration. Despite the relatively low dosage of the compound supplement in this study compared with that in previous research, it effectively reduced inflammatory markers and increased tear secretion. Notably, previous studies have failed to verify a relationship between DES severity and IL-1β concentration; nonetheless, this study established a correlation between increased tear secretion and decreased IL-1β levels.

After 12-week supplementation, the supplement group displayed a significant OSDI decrease of 6.6 points compared with the control group, corroborating the antioxidant and anti-inflammatory properties of fish oil, lutein, and zeaxanthin. DHA depletion is reportedly caused by H_2_O_2_, which also enhances lipid oxidation in retinal pigment epithelial cells; moreover, lutein and zeaxanthin enhance cellular defense against oxidative stress [52]. Studies have revealed that antioxidant supplementation may improve plasma antioxidant capacity, increase TBUT, and enhance tear secretion, thereby alleviating dry eye discomfort in patients with DES [53,54,55]. Similarly, this study observed a negative correlation between OSDI changes and plasma antioxidant capacity. A previous study involving a 12-week regimen of lutein (20 mg/day) and zeaxanthin (4 mg/day), including vitamin D and curcumin, yielded a 13-point reduction in the OSDI [31]. Another study involving EPA (360 mg/day) and DHA (240 mg/day) supplementation for 12 weeks demonstrated a significant 9.4-point reduction in the OSDI [56]. Our results align with these findings, showing a significant 14.6-point reduction in the OSDI, despite the lower dosages than those in previous studies.

DES owing to meibomian gland dysfunction is a clinical problem encountered in ophthalmology [57], and increased ROS levels can directly damage meibomian glands, leading to apoptosis [58], further reduced LLT, and increased tear evaporation, affecting TMH [59]. Previous studies have shown that the anti-inflammatory and antioxidant properties of fish oil, lutein, and zeaxanthin supplements can reduce eyelid inflammation [33]. We also observed a significantly positive correlation between MG loss and inflammation in tears. Several studies have demonstrated that varying dosages of EPA (1680–2000 mg/day) + DHA (560–1000 mg/day) for 12 weeks to 1 year significantly increase TBUT [60,61,62]. However, we found a significant increase in MG loss, a significant decrease in TBUT, and no significant changes in LLT and TMH in the supplement group. Meibomian gland function is easily influenced by external factors, such as screen time, sleep duration, environment, and ocular surgery [33]. Numerous studies confirm that ocular surgery reduces TBUT [63,64,65].

In this study, the supplement group had a significantly greater proportion of participants with a history of ocular surgery (66%) than the control group (26%). Additionally, the supplement group participants reported significantly less average daily sleep, potentially contributing to the observed lack of improvement. Moreover, the significant decrease in TBUT in the control group after 12 weeks might have been related to the significant increase in tear IL-17A levels; furthermore, we found a negative correlation between TBUT and IL-17A. IL-17A leads to ocular surface epithelial and tear function impairment [66,67,68] and stimulates MMPs, causing ocular surface damage and affecting tear film stability [69,70].

The strength of this study was its ability to successfully overcome challenges in collecting tear samples from patients with DES. This enabled a thorough investigation into tear antioxidant capacity, oxidative stress, and cytokine profiles. Notwithstanding, this study also had certain limitations. First, it was an open-label trial. This decision was based on previous studies indicating that using oil as a placebo may improve the tear film lipid layer and reduce ocular discomfort, thus impeding the elucidation of the effects of supplementation. To address this confounding factor, an open-label trial design was selected. Second, the supplement group had a significantly higher proportion of participants with a history of ocular surgery and a reduced sleep duration, which may have contributed to the observed increase in meibomian gland loss and the decrease in tear break-up time, potentially affecting the supplementation’s overall efficacy. Finally, this study involved a relatively small sample. Larger sample sizes will aid future studies in comprehensively elucidating the antioxidant effects of multicomponent supplements.

## 5. Conclusions

In conclusion, this study suggests that a 12-week supplementation regimen comprising EPA, DHA, lutein, and zeaxanthin has the ability to alleviate dry eye symptoms, enhance tear secretion, and mitigate the tear inflammatory response in patients with DES. However, a larger study is required to confirm these findings and provide more definitive recommendations for DES treatment.

## Figures and Tables

**Table 1:** Characteristics, visual outcome experiences, and dry eye questionnaires of study groups at baseline.

Variables ^1^	Control Group (*n* = 23)	Supplement Group (*n* = 29)	*p*-Value
Age (y)	56.48 ± 7.81	59.59 ± 10.33	0.24
Gender (M/F)	2/23	8/29	0.16
Age of menopause	50.23 ± 4.44	51.71 ± 6.06	0.15
Menopause (*n*, %)	12 (52%)	14 (48%)	0.1
High myopia (*n*, %)	7 (30%)	7 (24%)	0.85
High astigmatism (*n*, %)	4 (17%)	10 (34%)	0.29
Eye surgery experience (*n*, %)	6 (26%) *	19 (66%)	0.01
Symptoms of dry eye			
Dryness (*n*, %)	20 (87%)	22 (76%)	0.48
Fatigue (*n*, %)	20 (87%)	21 (72%)	0.31
Foreign body sensation (*n*, %)	14 (61%)	15 (52%)	0.71
Photophobia (*n*, %)	13 (57%)	17 (59%)	0.90
Itching (*n*, %)	11 (48%)	14 (48%)	0.81
Burning sensation (*n*, %)	4 (17%)	4 (14%)	1.00
Blurred or poor vision (*n*, %)	17 (74%)	22 (76%)	0.87
Pain (*n*, %)	5 (22%)	7 (24%)	0.90

^1^ Values are presented as mean ± SD. * Values are significantly different between control and supplement groups at baseline.

**Table 2:** Lifestyle habits of subjects from study groups at weeks 0 and 12.

Variables ^1^	Control Group (*n* = 23)		Supplement Group (*n* = 29)		Week 0	Week 12
	Week 0	Week 12	Week 0	Week 12	*p*-Value	
Anti-inflammatory drugs (*n*, %)						
No	20 (87%)	19 (90%)	28 (97%)	28 (100%)	0.31	0.18
Yes	3 (13%)	2 (10%)	1 (3%)	0 (0%)		
Frequency of using artificial tears (times, %)						
No	6 (26%)	5 (24%)	11 (38%)	11 (39%)	0.39	0.22
1–2	5 (22%)	6 (29%)	5 (17%)	5 (18%)		
3–4	11 (48%)	10 (47%)	8 (28%)	8 (29%)		
5–6	1 (4%)	0 (0%)	4 (14%)	2 (7%)		
>6	0 (0%)	0 (0%)	1 (3%)	2 (7%)		
Frequency of using video display units (hour) (*n*, %)						
0	0 (0%)	1 (5%)	0 (0%)	0 (0%)	0.14	0.45
2	5 (22%)	4 (19%)	4 (14%)	4 (15%)		
2–4	4 (17%)	3 (14%)	8 (28%)	6 (21%)		
4–6	5 (22%)	4 (19%)	1 (3%)	2 (7%)		
>6	9 (39%)	9 (43%)	16 (55%)	16 (57%)		
Frequency of reading (hour) (*n*, %)						
0	11 (48%)	14 (66%)	14 (49%)	15 (54%)	0.84	0.59
2	11 (48%)	6 (29%)	13 (45%)	12 (43%)		
2–4	0 (0%)	1 (5%)	1 (3%)	1 (3%)		
4–6	1 (4%)	0 (0%)	1 (3%)	0 (0%)		
>6	0 (0%)	0 (0%)	0 (0%)	0 (0%)		
Stay up late frequency (*n*, %)						
Never	7 (30%)	7 (33%)	10 (34%)	10 (36%)	0.85	0.32
Seldom	7 (30%)	8 (38%)	11 (38%)	12 (43%)		
Sometimes	5 (22%)	4 (19%)	4 (14%)	1 (3%)		
Often	3 (13%)	0 (0%)	2 (7%)	0 (0%)		
Always	1 (5%)	2 (10%)	2 (7%)	5 (18%)		
Average sleep time (hour) (*n*, %)						
<6	1 (4%)	2 (10%)	10 (35%)	5 (18%)	0.03	0.04
6–8	17 (74%)	15 (71%)	16 (55%)	23 (82%)		
>8	5 (22%)	4 (19%)	3 (10%)	0 (0%)		
Contact lens frequency (*n*, %)						
Never	23 (100%)	21 (100%)	27 (94%)	26 (93%)	0.44	0.50
Seldom	0 (0%)	0 (0%)	1 (3%)	0 (0%)		
Sometimes	0 (0%)	0 (0%)	1 (3%)	2 (7%)		
Often	0 (0%)	0 (0%)	0 (0%)	0 (0%)		
Always	0 (0%)	0 (0%)	0 (0%)	0 (0%)		

^1^ Values are presented as number with percentage.

**Table 3:** Ocular surface parameters of dry eye in study groups at weeks 0 and 12.

Variables ^1^	Control Group (*n* = 23)		Supplement Group (*n* = 29)	
	Week 0	Week 12	Week 0	Week 12
Schirmer test (mm)				
Right eye	6.17 ± 4.74	6.67 ± 5.94	5.93 ± 5.99	5.75 ± 4.32
Left eye	5.65 ± 5.11	5.38 ± 4.90	4.62 ± 4.79 ^b^	6.00 ± 4.55 ^a^
OSDI (score/100)	34.78 ± 12.32	33.43 ± 20.15 *	33.26 ± 16.04 ^a^	18.83 ± 15.19 ^b^
NIBUT (sec)				
Right eye	9.79 ± 2.94 ^a^	7.79 ± 2.21 ^b^	10.27 ± 4.67 ^a^	8.47 ± 1.96 ^b^
Left eye	9.07 ± 2.44	8.21 ± 2.15	10.63 ± 4.94	9.02 ± 3.00
TMH (mm)				
Right eye	0.21 ± 0.12	0.18 ± 0.07	0.18 ± 0.05	0.19 ± 0.07
Left eye	0.21 ± 0.08	0.19 ± 0.12	0.21 ± 0.06	0.20 ± 0.08
MG loss (%)				
Right eye	54.26 ± 15.45	52.81 ± 21.11	48.07 ± 18.97 ^b^	58.18 ± 20.83 ^a^
Left eye	50.22 ± 20.36	47.90 ± 15.14	48.07 ± 16.00	48.89 ± 17.05
LLT (0 of 6) right eye				
0 (*n*, %)	10 (43%)	13 (62%)	13 (45%)	15 (54%)
1 (*n*, %)	9 (39%)	6 (29%)	9 (31%)	8 (29%)
2 (*n*, %)	2 (9%)	2 (10%)	5 (17%)	5 (18%)
3 (*n*, %)	1 (4%)	0 (0%)	2 (7%)	0 (0%)
4 (*n*, %)	1 (4%)	0 (0%)	0 (0%)	0 (0%)
5 (*n*, %)	0 (0%)	0 (0%)	0 (0%)	0 (0%)
6 (*n*, %)	0 (0%)	0 (0%)	0 (0%)	0 (0%)
LLT (0 of 6) left eye				
0 (*n*, %)	9 (39%)	11 (52%)	8 (28%)	16 (57%)
1 (*n*, %)	8 (35%)	8 (38%)	15 (52%)	9 (32%)
2 (*n*, %)	5 (22%)	0 (0%)	5 (17%)	3 (11%)
3 (*n*, %)	1 (4%)	2 (10%)	1 (3%)	0 (0%)
4 (*n*, %)	0 (0%)	0 (0%)	0 (0%)	0 (0%)
5 (*n*, %)	0 (0%)	0 (0%)	0 (0%)	0 (0%)
6 (*n*, %)	0 (0%)	0 (0%)	0 (0%)	0 (0%)

^1^ Values are presented as mean ± SD and number with percentage; OSDI, ocular surface disease index; NIBUT, non-invasive tear break-up time; TMH, tear meniscus height; MG, meibomian gland; LLT, lipid layer thickness. ^a, b^ Values are significantly different between weeks 0 and 12 within the group; *p* < 0.05. * Values are significantly different between control and supplement groups at week 0 or 12; *p* < 0.05.

**Table 4:** Daily nutrient intakes of subjects from study groups at weeks 0 and 12.

Variables ^1^	Control Group (*n* = 23)		Supplement Group (*n* = 29)	
	Week 0	Week 12	Week 0	Week 12
Energy (Kcal/day)	1339.65 ± 352.82	1295.38 ± 399.70	1444.42 ± 489.84	1329.96 ± 465.37
Protein (g/day)	55.69 ± 32.76	65.87 ± 32.76	62.63 ± 32.76	56.97 ± 32.76
Protein (% total energy)	17.00 ± 4.00 ^b^	20.00 ± 5.00 ^a^	17.00 ± 5.00	17.00 ± 5.00
Carbohydrate (g/day)	147.07 ± 32.76	143.06 ± 32.76 *	188.15 ± 32.76 ^a^	149.21 ± 32.76 ^b^
Carbohydrate (% total energy)	44.00 ± 12.00 *	43.00 ± 12.00	52.00 ± 14.00	46.00 ± 12.00
Lipid (g/day)	54.41 ± 32.76	53.82 ± 32.76	50.66 ± 32.76	58.30 ± 32.76
Lipid (% total energy)	37.00 ± 11.00 *	39.00 ± 10.00	31.00 ± 10.00 ^b^	39.00 ± 10.00 ^a^
Saturated fatty acid (mg)	5294.55 ± 4932.79	4302.55 ± 3190.90	4516.58 ± 3700.62	7791.71 ± 7475.99
MUFA (mg)	5338.82 ± 4891.44	4985.13 ± 3283.96	4983.83 ± 4712.27	7572.83 ± 7478.71
PUFA (mg)	4793.86 ± 7681.52	7047.41 ± 5990.41	4981.86 ± 5060.43	4820.06 ± 6107.76
ALA (18:3) (mg)	494.98 ± 886.48	711.82 ± 672.75 *	441.21 ± 503.39	416.16 ± 628.13
EPA (20:5) (mg)	4.35 ± 7.64 ^b^	86.50 ± 259.19 ^a^	8.68 ± 15.72	22.05 ± 55.70
DHA (22:6) (mg)	14.66 ± 17.03 ^b^	50.26 ± 77.75 ^a^	19.87 ± 25.92	27.40 ± 48.41
S-EPA (20:5) (mg)	4.35 ± 7.64 ^b^	86.50 ± 259.19 ^a,^*	8.68 ± 15.72 ^b^	67.05 ± 55.70 ^a^
S-DHA (22:6) (mg)	14.66 ± 17.03 ^b^	50.26 ± 77.75 ^a,^*	19.87 ± 25.92 ^b^	57.40 ± 48.41 ^a^

^1^ Values are presented as mean ± SD. MUFA, monounsaturated fatty acid; PUFA, polyunsaturated fatty acid; ALA, α-linoleic acid; EPA, eicosapentaenoic acid; DHA, docosahexaenoic acid; S-EPA, includes dietary and supplement of eicosapentaenoic acid; S-DHA, includes dietary and supplement of docosahexaenoic acid. ^a, b^ Values are significantly different between weeks 0 and 12 within the group; *p* < 0.05. * Values are significantly different between control and supplement groups at week 0 or 12; *p* < 0.05.

**Table 5:** Antioxidant capacity, oxidative stress, and cytokines levels in study groups at weeks 0 and 12.

Variables ^1^	Control Group (*n* = 23)		Supplement Group (*n* = 29)	
	Week 0	Week 12	Week 0	Week 12
*Antioxidant capacities*				
Plasma TEAC (μmol/L)	4489.67 ± 280.44 ^a^ (4531.05)	4217.58 ± 233.51 ^b^ (4204.20)	4306.85 ± 397.63 (4345.32)	4315.88 ± 283.65 (4229.81)
Tear TEAC (μmol/μL)	11,242.65 ± 8775.13 (8281.86)	9772.83 ± 8014.59 (7924.94)	10,864.55 ± 6119.1 (10920.00)	8910.12 ± 4084.9 (8182.44)
*Oxidative stress*				
Plasma MDA (μmol/L)	1.02 ± 0.22 ^a^ (1.02)	0.87 ± 0.19 ^b^ (0.94)	1.10 ± 0.20 (1.06)	1.06 ± 0.26 (0.98)
Tear MDA (μmol/μL)	0.88 ± 0.99 ^b^ (0.51)	1.70 ± 1.45 ^a^ (1.28)	1.21 ± 1.22 (0.73)	1.37 ± 1.22 (1.21)
*Cytokines*				
IL 1β (*p*g/μL)	0.02 ± 0.05 (0.00)	0.02 ± 0.03 (0.01)	0.01 ± 0.01 (0.01)	0.01 ± 0.02 (0.01)
IL 2 (*p*g/μL)	0.01 ± 0.01 (0.01)	0.02 ± 0.02 (0.01)	0.02 ± 0.02 (0.01)	0.02 ± 0.02 (0.01)
IL 6 (*p*g/μL)	0.23 ± 0.60 (0.07)	0.26 ± 0.59 * (0.10)	0.17 ± 0.16 ^a^ (0.13)	0.10 ± 0.20 ^b^ (0.06)
IL 8 (*p*g/μL)	2.27 ± 3.35 * (0.49)	2.02 ± 2.90 (0.76)	3.75 ± 3.83 ^a^ (2.80)	1.57 ± 1.98 ^b^ (0.48)
IL 17A (*p*g/μL)	0.02 ± 0.01 ^b^ (0.01)	0.05 ± 0.06 ^a^ (0.02)	0.03 ± 0.04 (0.01)	0.03 ± 0.02 (0.02)
TNF-α (*p*g/μL)	0.11 ± 0.17 (0.05)	0.13 ± 0.18 (0.07)	0.08 ± 0.07 (0.05)	0.11 ± 0.1 (0.06)

^1^ Values are presented as mean ± SD (median). TEAC, Trolox equivalent antioxidant capacity. MDA, malondialdehyde. IL, interleukin. TNF-α, tumor necrosis factor-α. ^a, b^ Values are significantly different between weeks 0 and 12 within the group; *p* < 0.05. * Values are significantly different between control and supplement groups at week 0 or 12; *p* < 0.05.

**Table 6:** The correlation between the changes in ocular surface parameters of dry eye and changes in antioxidant capacity, oxidative stress, and cytokines levels ^1^.

Variables ^3^	Δ Schirmer Test		Δ NIBUT (sec)		Δ LLT (0 of 6)		Δ TMH (mm)		Δ MG Loss (%)		Δ OSDI
	Eye										
	Right	Left	Right	Left	Right	Left	Right	Left	Right	Left	
	*r* ^2^										
*Antioxidant capacities*											
Δ Plasma TEAC (μmol/L)	−0.03	0.02	−0.01	0.03	−0.14	−0.18	−0.14	−0.32 *	0.06	0.67	−0.30 *
Δ Tear TEAC (μmol/μL)	−0.72 *	−0.64 *	0.22	−0.17	−0.08	−0.18	0.17	−0.10	−0.14	0.23	−0.12
*Oxidative stress*											
Δ Plasma MDA (μmol/L)	0.18	−0.12	0.16	−0.05	0.01	0.07	0.02	0.22	0.05	−0.14	−0.04
Δ Tear MDA (μmol/μL)	−0.33 *	−0.23	0.28	−0.14	0.10	0.08	−0.06	0.06	0.20	−0.01	0.09
*Cytokines*											
Δ IL 1β (*p*g/μL)	−0.43 *	−0.25	−0.03	−0.06	−0.09	0.10	0.08	−0.08	−0.14	0.28	−0.06
Δ IL 2 (*p*g/μL)	−0.50 *	−0.48 *	0.12	−0.07	0.01	0.14	−0.10	−0.01	−0.13	−0.01	−0.06
Δ IL 6 (*p*g/μL)	−0.20	−0.26	0.16	0.05	0.09	0.23	0.09	0.12	0.01	0.44 *	−0.03
Δ IL 8 (*p*g/μL)	−0.33 *	−0.29 *	0.28	0.02	0.04	0.18	0.04	0.11	0.04	0.45 *	−0.16
Δ IL 17A (*p*g/μL)	−0.29	−0.27	−0.40 *	−0.26	−0.34	−0.29	−0.13	−0.41 *	−0.11	−0.02	0.05
Δ TNF-α (*p*g/μL)	−0.06 *	−0.56 *	0.30	0.04	−0.10	0.01	0.17	−0.11	−0.13	0.21	−0.22

^1^ NIBUT, non-invasive tear break-up time; LLT, lipid layer thickness; TMH, tear meniscus height; MG, meibomian gland. OSDI, ocular surface disease index. TEAC, Trolox equivalent antioxidant capacity. MDA, malondialdehyde. IL, interleukin. TNF-α, tumor necrosis factor-α. ^2^ *r*, correlation coefficient. ^3^ Variables are changes at weeks 0 and 12 [Δ (week 12-0)] in both groups. * Values are significant correlations between the changes in all parameters, *p* < 0.05.

**Table 7:** Multiple linear regression analysis of treatment with the changes in ocular surface parameters, oxidative stress, antioxidant capacities, and inflammatory indicators of dry eye in patents with dry eye syndrome.

Dependent Variables ^3^	Eye	Model 1 ^1^		Model 2 ^1^	
		β ^2^	SE ^2^	β ^2^	SE ^2^
Δ Schirmer test (mm)	Right	−1.10	1.94	−1.20	1.97
	Left	1.10	1.19	1.00	1.21
Δ NIBUT (sec)	Right	0.05	1.26	0.04	1.28
	Left	−0.89	1.07	−0.85	1.09
Δ LLT (0 of 6)	Right	0.19	0.21	0.18	0.21
	Left	−0.13	0.19	−0.11	0.19
Δ TMH (mm)	Right	0.02	0.03	0.03	0.03
	Left	0.01	0.03	0.01	0.03
Δ MG loss (%)	Right	11.14	0.04	10.63	5.69
	Left	3.05	6.20	2.55	6.28
Δ OSDI (score/100)		−6.38 *	2.49	−6.60 *	2.52
Δ Plasma TEAC (μM)		239.32 *	114.57	221.40	114.48
Δ Tear TEAC (*p*g/μL)		−21.93	2383.89	14.60	2426.32
Δ Plasma MDA (μM)		0.05	0.07	0.06	0.07
Δ Tear MDA (*p*g/μL)		−0.56	0.51	−0.61	0.53
Δ IL 1β (*p*g/μL)		0.01	0.01	0.01	0.01
Δ IL 2 (*p*g/μL)		−0.01	0.01	−0.01	0.01
Δ IL 6 (*p*g/μL)		−0.08	0.07	−0.01	0.07
Δ IL 8 (*p*g/μL)		0.25	1.93	0.22	1.96
Δ IL 17A (*p*g/μL)		−0.03	0.02	−0.03	0.02
Δ TNF-a (*p*g/μL)		0.01	0.05	0.01	0.05

^1^ Model 1 was not adjusted; model 2 was adjusted for age, gender, and menopause. ^2^ β, regression; SE, standard error, * *p* < 0.05. NIBUT, non-invasive tear break-up time; LLT, lipid layer thickness; TMH, tear meniscus height; MG, meibomian gland. OSDI, ocular surface disease index. TEAC, Trolox equivalent antioxidant capacity. MDA, malondialdehyde. IL, interleukin, TNF-α, tumor necrosis factor-α. ^3^ Variables are changes at weeks 0 and 12 [Δ (week 12-0)] in both groups.

## Data Availability

Data collected from human participants, as described in Table 1, are not available for confidentiality reasons.
